# Isolated benign persistent proteinuria with novel association of *CUBN* (cubilin) variants

**DOI:** 10.1002/ccr3.7502

**Published:** 2023-06-12

**Authors:** Vivian Shi, Quinn Stein, Dinah Clark, Sumit Punj, Robin Kremsdorf, Mohammed Faizan

**Affiliations:** ^1^ Warren Alpert Medical School of Brown University Rhode Island Providence USA; ^2^ Department of Medicine, Rhode Island Hospital Rhode Island Providence USA; ^3^ Department of Pediatrics, Hasbro Children’s Hospital Rhode Island Providence USA; ^4^ Natera, Inc. Texas Austin USA

**Keywords:** cubilin gene, genetic testing, kidney, monogenic, persistent proteinuria

## Abstract

We present two siblings with persistent proteinuria and normal kidney function, each carrying the same compound heterozygous variants in the *CUBN* gene. The *CUBN*‐related phenotype appears to be dependent upon both variant type and the domain site within the gene. Knowledge of *CUBN* status may allow for avoidance of invasive testing.

## 1 INTRODUCTION

Persistent proteinuria is a reliable predictor for progressive kidney and cardiovascular disease.^1^ In healthy kidneys, up to 3 g of albumin are freely filtered daily from the glomerulus to be recaptured in the proximal tubule. Defects in this process that result in proteinuria can be divided into three subcategories – glomerular, overflow, and tubular.^2^ Glomerular proteinuria results from increased permeability of the glomerular capillary wall resulting in abnormal passage of proteins from the glomerulus. Overflow proteinuria describes an overproduction of an individual protein, as occurs in multiple myeloma. Tubular proteinuria occurs due to increased urinary loss of low molecular weight proteins with a classic example being Fanconi syndrome.^3^, ^4^, ^5^ Improved access to genetic testing has enabled identification of monogenic causes of proteinuria, helping to direct therapy for individual patients.^4^ In one international study utilizing exon sequencing, single‐gene causes of steroid‐resistant nephrotic syndrome, in which proteinuria is a primary presentation, were detected in 29.5% (526/1783) of families where symptoms manifested in individuals younger than 25 years of age.^4^

**FIGURE 1:**
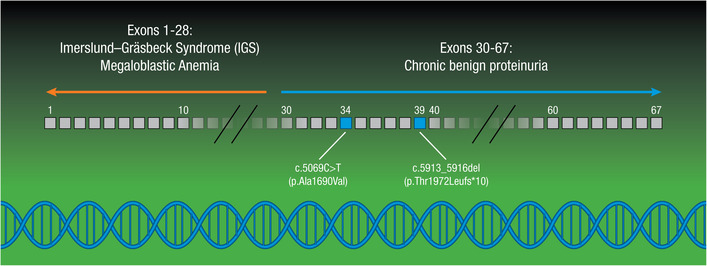
Schematic of *CUBN* gene and exons, adapted from Domingo‐Gallego et al.^2^ Previously reported variants in exons 1–28 previously have been associated with IGS with megaloblastic anemia.^2^ Variants in exons 29–67, the region encompassing c.5913_5916del (p.Thr1972Leufs*10) and c.5069C > T (p.Ala1690Val) which were reported here, have been associated with chronic benign proteinuria.^2^, ^8^, ^9^, ^10^, ^11^, ^12^

The *CUBN* gene, which encodes the cubilin protein, was first identified as a monogenic cause of tubular proteinuria in 2011 by Ovunc et al.^6^ Cubilin is a multiligand receptor that acts in concert with two transmembrane proteins, megalin and amnionless (AMN), to anchor to cell membranes. This interaction facilitates intracellular transport of cubilin–ligand complexes.^2^, ^7^, ^8^, ^9^ These proteins have limited expression in the small intestines and proximal tubules of the kidney. Defects in cubilin were previously only known to result in an autosomal recessive condition called Imerslund–Gräsbeck syndrome (IGS), which is characterized by intestinal vitamin B12 malabsorption, megaloblastic anemia, and, in half of cases, proteinuria.^10^ *CUBN* variants noted in cases of IGS affect the N‐terminal half of the encoded protein, which contains a domain responsible for vitamin B12 binding (Figure 1).^2^, ^10^, ^11^, ^12^ In cases of renally isolated disease, *CUBN* variants affect amino acid sequence downstream of the B12 binding‐domain, suggesting that more C‐terminal domains are crucial for urinary protein reabsorption, predominantly albumin (Figure 1).^2^, ^5^, ^9^, ^10^, ^11^, ^12^

Existing literature is limited regarding the clinical manifestations and outcomes of isolated proteinuria associated with *CUBN* variants. The first known cases were two children born to consanguineous parents with homozygous deleterious frameshift variants in *CUBN*.^7^ These individuals had documented benign proteinuria with normal kidney function in the absence of megaloblastic anemia.^7^ A second report showed homozygous stop‐gain variants and similar symptoms of isolated proteinuria in another set of siblings (also born to consanguineous parents).^4^ Additional pathogenic variants in *CUBN* have been identified via cohorts of patients with chronic proteinuria and normal kidney function. Patient cohorts studied by Bedin et al. share similar characteristics of chronic proteinuria with onset in childhood (average age of onset 10.9 years), normal kidney histopathology and kidney function as measured by eGFR.^9^ These patients continued to have normal kidney function at average age of 17 with limited longer‐term follow‐up and showed lack of proteinuria‐lowering effects with ACE inhibitors.^9^ In this paper, we report the identification of biallelic *CUBN* variants in a young non‐consanguineous sibling pair with isolated chronic proteinuria and normal kidney function.

## 2 CASE PRESENTATION

**FIGURE 2:**
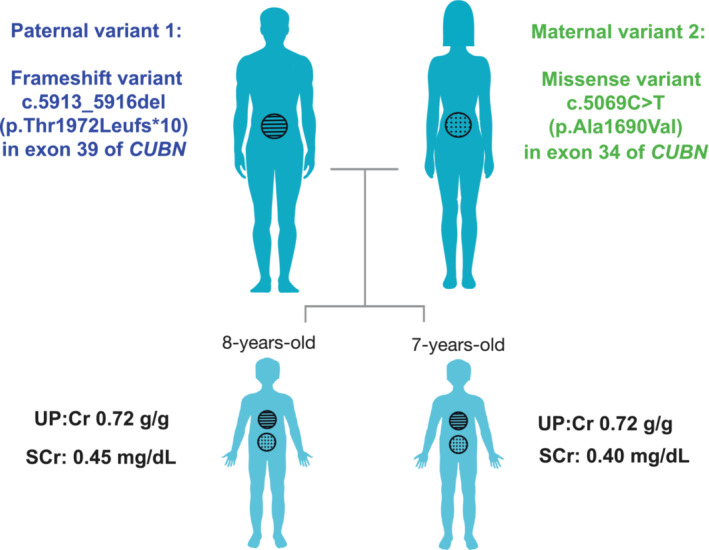
A cartoon illustration showing *CUBN* variants inherited from each parent (depicted by circles filled with dots or lines) in the proband and his sibling.

An 8‐year‐old male was referred to pediatric nephrology for evaluation of subnephrotic range proteinuria. Workup found normal serum creatinine (0.45 mg/dL), normal complement levels (C3/C4), negative ANCA and ANA, and normal kidney ultrasound. The patient's younger brother was also later diagnosed with isolated, persistent subnephrotic range proteinuria at age 7, with normal serum creatinine (0.44 mg/dL) and complement levels. Neither sibling had evidence of megaloblastic anemia nor family history to suggest IGS. Parents declined kidney biopsy for both children and the sibling pair were maintained on low‐dose lisinopril. Both siblings had a slight reduction in proteinuria measured by urine protein/creatinine ratio (UP:Cr) averaged over the two most recent values compared to baseline value. After 7 years of follow‐up, reduction in proteinuria was 15% (UP:Cr 0.72 vs. 0.61 g/g) and 17% (UP:Cr 0.72 vs. 0.60 g/g) in the older and younger siblings, respectively (Figure 2).

Genetic testing via a 382 gene kidney disease panel (the Renasight™ test)^13^ in both siblings identified a pair of likely pathogenic variants in *CUBN*: a paternally inherited frameshift variant, c.5913_5916del (p.Thr1972Leufs*10) in exon 39, and a maternally inherited novel missense variant, c.5069C > T (p.Ala1690Val) in exon 34. Parental history was negative for kidney disease including proteinuria. Genetic testing of the parents showed that each was a heterozygous carrier, confirming biallelic inheritance in both children (Figure 2). The identified variants affect the C‐terminal half of the *CUBN* protein, similar to previous case reports of *CUBN* variants that document an association with benign chronic subnephrotic proteinuria in the absence of IGS (Figure 1).^7^ Given that the variants were confirmed to be in *trans* and the children's clinical presentation aligned with *CUBN*‐related disease, a molecular etiology was confirmed for this family.

## 3 DISCUSSION

Few cases of benign isolated subnephrotic proteinuria in association with C‐terminal cubilin defects have been described in the literature. The spectrum of clinical presentations associated with *CUBN* pathogenic variants ranges from megaloblastic anemia type 1 (IGS) to isolated benign proteinuria.^2^, ^7^, ^8^, ^9^, ^10^ The phenotype appears to be dependent on the site of the pathogenic variant within the *CUBN* gene and the loss of function of certain domains.^2^, ^10^, ^11^, ^12^ We present a unique case of non‐consanguineous siblings with compound heterozygous variants associated with isolated chronic proteinuria. Based on family history, our findings are consistent with those of previous reports for an autosomal recessive inheritance pattern and provide additional support for the gene–disease association.

Our case highlights the early application of comprehensive genetic screening for diagnostic workup of pediatric‐onset proteinuria, minimizing the need for further invasive testing such as kidney biopsy. Since the specific variants and their location can predict phenotype,^2^, ^10^, ^11^, ^12^ we were able to further classify the disease as likely chronic benign proteinuria by recognizing that these variants reside in exons 34 and 39. Based on the genetic findings and clinical correlation with limited previous *CUBN* case reports, it appears the siblings have a good long‐term prognosis of likely benign proteinuria, having already had 7 years of clinical follow‐up without signs of progressive kidney disease. A previous sibling cohort started on RAAS blockade had a similarly mild reduction in proteinuria and stable normal kidney function at 4‐year follow‐up.^4^ Importantly, the oldest patient reported in the literature with biallelic *CUBN* pathogenic variants and chronic proteinuria was documented to have normal kidney function at 66 years of age.^5^ While existing case reports and cross‐sectional studies postulate benign clinical courses, the long‐term outcomes of these few reported patients are not definitively known. Of note, analysis of participants from second and third generations of the original Framingham Heart Study with microalbuminuria did not show any significant association between a specific *CUBN* risk allele (missense SNP rs1801239, 12984V, Ile‐>Val) and cardiovascular disease.^14^ At present, current evidence suggests prolonged normal kidney function in individuals with proteinuria resulting from certain *CUBN* variants. Altogether, identification of *CUBN* variants through genetic testing can provide important information about patient disease course. Future studies of these rare patients are necessary to inform long‐term outcome, prognosis, and management, as well as uncovering possible therapeutic interventions.

## AUTHOR CONTRIBUTIONS

**Vivian Shi:** Conceptualization; investigation; resources; visualization; writing – original draft; writing – review and editing. **Quinn Stein:** Conceptualization; investigation; project administration; resources; writing – original draft; writing – review and editing. **Dinah Clark:** Data curation; investigation; methodology; resources; writing – original draft; writing – review and editing. **Sumit Punj:** Formal analysis; methodology; resources; writing – original draft; writing – review and editing. **Robin Kremsdorf:** Conceptualization; writing – original draft; writing – review and editing. **Mohammed Faizan:** Conceptualization; investigation; supervision; visualization; writing – original draft; writing – review and editing.

## FUNDING INFORMATION

No funding was received for this study.

## CONFLICT OF INTEREST STATEMENT

QS, DC, and SP are full‐time employees at Natera Inc. with stocks or options to own stocks in the company. VS, RK, and MF declare no conflicts of interest.

## CONSENT

Written informed consent to publish was obtained from the parents of the siblings described in this case report.

## Data Availability

The data generated and/or analyzed during the current study are not publicly available due to privacy/ethical restrictions but are available from the corresponding author on reasonable request.
